# Minutes of the Regular Monthly Meeting of the Western Texas Medical Association, Held in San Antonio, Texas, March 30, 1899

**Published:** 1899-04

**Authors:** 


### Society Notes.

### Minutes of the Regular Monthly Meeting of the Western Texas Medical Association, held in San Antonio, Texas, March 30, 1899.

Dr. John S. Lankford in the chair, Dr. William E. Luter, Secretary.

The members present were: Doctors C. E. R. King, B. F. Kingsley, E. Hertzberg, John S. Lankford, Robert E. Moss, William F. James, S. Burg, H. A. Tutweiler, E. H. Souvignet, John V. Spring and William E. Luter.

Visitors present were: Doctors J. H. Moore, John T. Harrison, H. A. Blair and — Brown.

Minutes of the previous regular meeting were read and approved.

The applications for membership of Doctors J. H. Moore, H. A. Blair and Robert Dinwiddie were presented and referred to the Board of Censors.

Committee (Dr. Paschal, chairman) appointed to see the city council with reference to giving physicians right of way on our streets continued.

Also committee (Dr. Spring, chairman) with reference to resolutions regarding the revision in our text-books continued.

The report of the committee apointed by the chair to confer with the committee on the revision of our city charter was read by the committee as follows:

"Mr. President: Your committee appointed to formulate some suggestions relative to the betterment of the health and sanitary matters of this city, and as expressive of the views of the medical fraternity, beg, through their chairman, to submit the following points, which they hope may be met with your endorsement, and be incorporated into the charter now undergoing revision:

"First. That provision be made for a health board, clearly defining the powers of said board.

"Second. That the health officer be elected by the people at the time of the election of school commissioners.

"Third. That the mayor and health officer appoint the board.

"Fourth. The health officer to be chairman of the said board of health."

Dr. King moved that a clause be added to Dr. Kingsley's report defining the authority and jurisdiction of the board of health and city health officer, and, after considerable discussion, the motion was accepted.

A motion to acept the committee's report as amended was made, seconded and carried, Drs. Souvignet and Moss opposing.

Dr. Tutweiler offered the following resolution:

"*Resolved,* That the Western Texas Medical Association earnestly protests against the proposed bill charging board to the officers of the asylums during their term of office, believing that it is against the best interests of the State and of these institutions, and we respectfully request our representatives to oppose said bill in every way possible."

It was moved that the Secretary be instructed to communicate the above resolution to the Legislature for their consideration.

Motion carried.

A resolution was offered by Dr. Burg as follows:

"*Resolved,* That it is the sense of this Association that the ordinance constituting a board of health to serve the city gratitous is incorrect in its principle, and we regard it an injustice to the profession to consider the labor, time and knowledge of the physicians used for the benefit of the city as worth nothing."

By motion the resolution was adopted.

The following resolution was offered by Dr. Tutweiler, and by unanimous vote carried:

"WHEREAS, Providence has taken from us our friend and co-laborer, Dr. Howard, of Devine, Texas; be it

"*Resolved,* By the Western Texas Medical Association, that in the death of Dr. Howard our society has lost a valuable member; that we deeply sympathize with his family; that a copy of this resolution be spread upon our minutes, and copies sent to our daily papers and medical journal, and also to his family."

A resolution offered by Dr. Kingsley reads as follows:

"WHEREAS, Dr. B. E. Hadra has retired from the office of city physician, be it

"*Resolved* by this Association, That we acknowledge the ability and energy with which Dr. Hadra has conducted the health affairs of this city during the past two years."

Upon motion, the above resolution was unanimously carried.

The hour being late, and the members on program absent, a motion by Dr. Burg to adjourn this meeting until Thursday, April 20, 1899, was carried.

JNO. S. LANKFORD, President.

WM. E. LUTER, Secretary and Treasurer.

